# Improved Glycaemia Correlates with Liver Fat Reduction in Obese, Type 2 Diabetes, Patients Given Glucagon-Like Peptide-1 (GLP-1) Receptor Agonists

**DOI:** 10.1371/journal.pone.0050117

**Published:** 2012-12-06

**Authors:** Daniel J. Cuthbertson, Andrew Irwin, Chris J. Gardner, Christina Daousi, Tej Purewal, Niall Furlong, Niru Goenka, E. Louise Thomas, Valerie L. Adams, Sudeep P. Pushpakom, Munir Pirmohamed, Graham J. Kemp

**Affiliations:** 1 Department of Obesity and Endocrinology, University Hospital Aintree, Liverpool, Liverpool, United Kingdom; 2 Department of Obesity and Endocrinology, Institute of Ageing and Chronic Disease, University of Liverpool, Liverpool, Liverpool, United Kingdom; 3 Department of Diabetes and Endocrinology, Royal Liverpool University Hospital, Liverpool, United Kingdom; 4 Department of Diabetes and Endocrinology, St Helen’s and Knowsley, Whiston, Whiston, United Kingdom; 5 Department of Diabetes and Endocrinology, Countess of Chester Hospital, Chester, United Kingdom; 6 Metabolic and Molecular Imaging Group, MRC Clinical Sciences Centre, Imperial College London, London, United Kingdom; 7 Magnetic Resonance and Image Analysis Research Centre, University of Liverpool, Liverpool, United Kingdom; 8 The Wolfson Centre for Personalised Medicine, Department of Molecular and Clinical Pharmacology, Institute of Translational Medicine, University of Liverpool, Liverpool, United Kingdom; 9 Department of Musculoskeletal Biology, Institute of Ageing and Chronic Disease, University of Liverpool, Liverpool, United Kingdom; Universita Magna-Graecia di Catanzaro, Italy

## Abstract

Glucagon-like peptide-1 receptor agonists (GLP-1 RA) are effective for obese patients with type 2 diabetes mellitus (T2DM) because they concomitantly target obesity and dysglycaemia. Considering the high prevalence of non-alcoholic fatty liver disease (NAFLD) in patients with T2DM, we determined the impact of 6 months’ GLP-1 RA therapy on intrahepatic lipid (IHL) in obese, T2DM patients with hepatic steatosis, and evaluated the inter-relationship between changes in IHL with those in glycosylated haemoglobin (HbA_1_c), body weight, and volume of abdominal visceral and subcutaneous adipose tissue (VAT and SAT). We prospectively studied 25 (12 male) patients, age 50±10 years, BMI 38.4±5.6 kg/m^2^ (mean ± SD) with baseline IHL of 28.2% (16.5 to 43.1%) and HbA_1_c of 9.6% (7.9 to 10.7%) (median and interquartile range). Patients treated with metformin and sulphonylureas/DPP-IV inhibitors were given 6 months GLP-1 RA (exenatide, n = 19; liraglutide, n = 6). IHL was quantified by liver proton magnetic resonance spectroscopy (^1^H MRS) and VAT and SAT by whole body magnetic resonance imaging (MRI). Treatment was associated with mean weight loss of 5.0 kg (95% CI 3.5,6.5 kg), mean HbA_1c_ reduction of 1·6% (17 mmol/mol) (0·8,2·4%) and a 42% relative reduction in IHL (−59.3, −16.5%). The relative reduction in IHL correlated with that in HbA_1_c (ρ = 0.49; p = 0.01) but was not significantly correlated with that in total body weight, VAT or SAT. The greatest IHL reduction occurred in individuals with highest pre-treatment levels. Mechanistic studies are needed to determine potential direct effects of GLP-1 RA on human liver lipid metabolism.

## Introduction

The glucagon-like peptide-1 (GLP-1) receptor agonists are used as blood glucose-lowering treatments of obese patients with type 2 diabetes mellitus (T2DM). GLP-1 acts through several distinct mechanisms including stimulation of glucose-dependent insulin secretion, inhibition of glucagon secretion, delay of gastric emptying and promotion of weight loss through central inhibition of appetite [1]. In patients treated with GLP-1 receptor agonists (GLP-1 RA), the weight loss is predominantly associated with a reduction in adipose tissue [2] although the inter-relationships between the relative amounts of visceral, subcutaneous and hepatic fat loss remain unknown.

NAFLD describes a disease spectrum with excessive deposition of fat within the liver (hepatic steatosis), which may be associated with inflammation, cell death, and fibrosis (non-alcoholic steatohepatitis, NASH), ultimately progressing to cirrhosis [3]. NAFLD has a high prevalence in patients with type 2 diabetes, diagnosed variously on the basis of abnormal liver biochemistry, ultrasonography [4], proton magnetic resonance spectroscopy (^1^H MRS) or liver biopsy [5]–[7]. For example, the Edinburgh Diabetes Study, which carefully excluded secondary causes of steatosis, demonstrated hepatic steatosis in 57% of type 2 diabetes patients by ultrasonography, with NAFLD-related steatosis in 43% [4]. This high prevalence is not simply explained by the high incidence of obesity in type 2 diabetes, as liver fat is increased in patients with type 2 diabetes compared with age- and BMI-matched healthy controls [5], [8]–[11] but is important for several reasons. Epidemiological studies highlight an increased prevalence of chronic liver disease and hepatocellular carcinoma in patients with type 2 diabetes [10], [12]. Furthermore, NAFLD is associated with a higher prevalence of cardiovascular disease and a greater burden of diabetic complications in patients with type 2 diabetes [13]–[16]. The impact of reducing liver fat by lifestyle or pharmacological intervention on the natural history or the frequency of these long-term complications is unknown.

Current treatment of NAFLD is aimed at weight loss through lifestyle interventions involving diet and exercise [17]–[20]. Petersen *et al.* demonstrated that modest weight reduction (∼8 kg) in patients with type 2 diabetes substantially reduced hepatic steatosis (by ∼80%) and concomitantly improved hepatic insulin resistance [19], [21]. Treatment with metformin or glitazone (either rosiglitazone or pioglitazone) may also ameliorate NAFLD, with metabolic and histological improvement, predominantly through reduced steatosis and inflammation, with little effect on fibrosis [22]–[25]. As for GLP-1 RAs, Buse *et al* demonstrated that 2 years exenatide therapy was associated with significant improvement in abnormal liver transaminases, biomarkers of hepatocyte injury most commonly reflecting NAFLD [26]. Tushuizen directly examined the effect of exenatide therapy on hepatic steatosis measured non-invasively by ^1^H MRS, showing that 44 weeks of treatment was associated with a reduction in liver fat from 16.0 to 5·4% [27]. This is supported by a recent case series of 8 patients with type 2 diabetes and biopsy-proven NAFLD who underwent liver biopsies before and after 28 weeks of exenatide therapy, in 3 of whom liver histology improved [28].

The present study was undertaken to define the inter-relationship between the reduction in intrahepatic lipid (IHL) and changes in glycaemic control and body composition (body weight, VAT and SAT) in patients with type 2 diabetes and hepatic steatosis receiving GLP-1 receptor agonists, to determine what factors are associated with the reduction in liver fat observed with these drugs.

## Research Design and Methods

### Inclusion and Exclusion Criteria

Patients were prospectively recruited from four specialist diabetes outpatient clinics. The inclusion criteria were: i) known type 2 diabetes with ii) obesity (BMI>35 kg/m^2^), and iii) at least 3 months treatment on a stable dose regime of maximal doses of metformin, with either sulphonylureas or DPP-IV inhibitors. Exclusion criteria were: i) treatment within the last 3 months with pioglitazone, orlistat, insulin, any other drugs associated with hepatic steatosis (including glucocorticoids, tamoxifen, amiodarone or methotrexate), ii) weekly alcohol intake >14 units for women or >21 units for men, iii) any history of liver disease including metabolic or auto-immune liver disease or viral hepatitis. Patients who fulfilled these criteria were screened for hepatic steatosis using ^1^H MRS and those with hepatic steatosis (IHL>5.5%) continued in the study. A pre-existing clinical diagnosis of NAFLD was not deemed necessary for inclusion due to the high prevalence of undiagnosed NAFLD in T2DM. The Liverpool Research Ethics Committee approved the study protocol and all participants gave their written informed consent.

25 of the 31 eligible patients who were underwent baseline scanning completed the study. The six dropouts initiated treatment after baseline assessments but one declined follow-up, two developed unrelated medical issues requiring withdrawal from study and three discontinued GLP-1 treatment due to gastrointestinal side effects.

### Drug history of patients

All patients were treated with the maximal tolerated dose of metformin for a minimum of 3 months before recruitment and they remained on the same mean metformin dose throughout (4 patients required a minor dosage adjustment). 16 patients were treated with sulphonylurea (SU) therapy with gliclazide (n = 12; continued in all patients with adjustment of dose in 4) or with the DPP-IV inhibitor, sitagliptin (n = 4; discontinued in all). No patients had received pioglitazone within the 3 months prior to recruitment.

### Initiation of GLP-1 RA Therapy

Participants were initiated on GLP-1 RA therapy in accordance with UK National Institute for Clinical Excellence guidelines [29] which mandate that only selected patients are eligible for GLP-1 RA therapy: those patients with a BMI>35 kg/m^2^, and those with a BMI<35 kg/m^2^ in whom there is a co-morbid condition for which weight loss would be desirable, e.g. obstructive sleep apnoea, NAFLD or polycystic ovary syndrome. The choice of GLP-1 RA was in accordance with NICE recommendations at the time of drug initiation (exenatide was approved and available for use in UK by NICE before liraglutide was approved). Of the patients who completed the 6-month study period, 19 were treated with exenatide and 6 with liraglutide. Exenatide was initiated at 5 mcg twice daily, titrated to 10 mcg twice daily after one month; liraglutide was initiated at 0·6 mg once daily, titrated to 1·2 mg once daily. All patients remained under the supervision of a diabetes specialist team throughout the study.

### Lifestyle Variables

Patients were not provided with specific guidance on modification of diet or physical activity. 18 patients were abstinent from alcohol throughout; mean weekly consumption in the remaining patients was 3±5 units pre-treatment and 2±5 units post-treatment. 24 patients were non-smokers throughout the study. All measurements were made at baseline, prior to initiation of the drug, and after 6 months of therapy.

### Determination of Body Composition

A single observer (AI), who was not involved in the clinical care of the patients, made all of the measurements. Body weight was measured with a Tanita bioimpedance analyser, wearing light clothing (Tanita BC420, Dolby Medical, Stirling, UK). Height was measured using a stadiometer to the nearest 0.5 cm (Seca, Birmingham, UK). Waist circumference was taken at the mid-point between the anterior superior iliac spine and the lower edge of the rib cage.

### Serum Biochemistry and Total Adiponectin Concentration

Serum glucose, lipid profiles and liver biochemistry were determined by using the Olympus AU2700 analyser (Beckman Coulter (UK) Ltd) with standard proprietary reagents. Total serum adiponectin levels were determined using a quantitative sandwich ELISA kit (R & D Systems Europe Ltd, Oxon, UK). Each sample was run in triplicates and the mean value was obtained by calculation using the standard curve method. Paired pre- and post-treatment samples were run on the same ELISA plate to minimise plate-related assay variation. The percentage inter-assay variation was between 0.4–9.8%.

### Magnetic Resonance Methods

Participants underwent MR scanning in a 1.5T Siemens Symphony scanner (Siemens Medical Solutions, Erlangen, Germany) at a single site, the University of Liverpool Magnetic Resonance and Image Analysis Research Centre, as previously described [30] A single experienced radiographer (VLA) performed all scans.

### Volumetric Analysis of Subcutaneous and Visceral Fat

Abdominal subcutaneous adipose tissue (abdominal SAT) and abdominal visceral adipose tissue (abdominal VAT) were calculated from whole body axial T1-weighted fast spin echo scans (axial scans, 10 mm slice thickness followed by a 10 mm gap using the integral body coil). All images were anonymised and blinded to time-point, but not to subject (to facilitate matching anatomical landmarks), and analysed by Vardis (Vardis Group Inc., London, UK) using SliceOMatic (Tomovision, Montreal, Canada).

### Proton Magnetic Resonance Spectroscopy

(^1^H MRS): In *liver*, NAFLD was defined as intrahepatocellular lipid (IHL) >5·5% measured by ^1^H MRS [7] Three voxels of interest were identified in the liver standard sites avoiding ducts and vasculature. In *skeletal muscle,* ^1^H MRS was used to measure intramyocellular lipid (IMCL), using a single voxel in each of the tibialis anterior (TA) and soleus (Sol) muscles, avoiding bone, fascia and the neurovascular bundle. Single voxel spectroscopy was conducted at each of these five sites. Voxel size was 20×20×20 mm, TE 135 ms, TR 1500 ms, with 64 acquisitions. Where the musculature was too small to allow placement of a 20 mm voxel, a 15×15×20 mm voxel was placed and the number of acquisitions increased to 200 to maintain signal-to-noise ratio. In both liver and muscle, voxel placement in post-treatment studies was guided by reference to the pre-treatment images. ^1^H MR spectra were quantified using the AMARES algorithm in the software package jMRUI-3.0. As previously described, IHCL is expressed as percentage of CH_2_ lipid signal amplitude relative to water signal amplitude after correcting for T_1_ and T_2_, and IMCL is expressed as CH_2_ lipid amplitude relative to total creatine amplitude after correcting for T_1_ and T_2_ [31]). Fat quantification by ^1^H MRS has been validated against gold standard biochemical measurements [32].

### Reproducibility of MRI and MRS Analysis

*Volumetric analysis of adipose tissue content:* the mean coefficient of variations (CoV) were determined as total body fat, 1–2%; total subcutaneous fat, 3–4%*;* abdominal subcutaneous fat, 1–3%; visceral fat, 6–8%.

### Quantification of Liver Fat (IHCL CH_2_/Water)

The mean inter-examination CoV for using this protocol is 7% (range 4–12%) and the mean intra-examination CoV is 6% [31].

### Statistical Analysis

Statistical analyses were performed using Stata/IC 12.0 software [StataCorp LP, College Station, TX, USA]. Demographic data are presented as mean ± standard deviation except where distributions were non-normal, in which case median and interquartile range (IQR) are presented. Paired t-tests were used to assess the absolute differences between individuals pre- and post-treatment (pre – post) and are presented as mean differences with 95% confidence intervals. In some cases log transformation of the data was necessary to achieve normality and where unsuccessful the Wilcoxon matched-pairs test was used. Relative changes are presented as median and interquartile range. Subgroups were compared using unpaired t-tests. Correlation was assessed using the Pearson correlation coefficient (r) where the relevant assumptions were met; otherwise the Spearman rank correlation coefficient (ρ) was used. Linear regression was used to assess the linear relationships between IHCL changes and changes in other variables independently. No corrections have been made for multiple comparisons.

## Results

### Baseline Characteristics (Table 1)

25 patients (12 male), age 50±10 years, BMI 38.4±5.6 kg/m^2^ with a duration of T2DM of 6±4 years (means ± SD) participated. Baseline median liver fat was 28.2% (IQR 16.5, 43.1).

**Table 1:** Clinical, biochemical, metabolic and body composition characteristics before and after 6 months of treatment with GLP-1 analogues.

Variable	Pre-treatment	Post-treatment	Change (95% confidence interval)	Median Relative change % (Interquartile range)	p value
**Clinical characteristics**					
Body weight (kg)	116.7 (102.4 to 125.3)	111.3 (98.3 to 118.7)	−5 (−6.5, −3.5)	−4.3 (−6.4 to −1.8)	<0.00005
BMI (kg/m^2^)	38.4 (5.6)	36.7 (5)	−1.7 (−2.2, −1.2)	−4.5 (−6.4 to −1.9)	<0.00005
Waist circumference (cm)	125 (111 to 132)	120 (107 to 128)	−5.2 (−7.4, −2.9)	−3.2 (−7.9 to −1.1)	0.0001
Fat (L)	21 (5.1)	19.3 (4.8)	−1.8 (−2.5, −1.1)	−7.5 (−11.7 to 0.3)	<0.00005
Systolic BP (mmHg)	133 (120 to 145)	125 (120 to 138)	–	−6.1 (−11.3 to 0)	0.0151
Diastolic BP (mmHg)	84 (78 to 89)	80 (75 to 84)	–	−5.9 (−10.5 to 0)	0.0062
**Biochemical and metabolic**					
FPG (mmol/l)	10.8 (8.9 to 14)	8.3 (6 to 10.1)	–	–	0.0007
HbA_1_c (%)	9.6 (7.9 to 10.7)	7.5 (6.5 to 8.5)	−1.6 (−2.4, −0.8)	–	0.0003
AST (U/ml)	27 (20 to 38)	25 (21 to 33)	–	–	0.0998
ALT (U/ml)	40 (31 to 44)	31 (27 to 43)	–	–	0.0118
GGT (U/ml)	69 (34 to 100)	43 (22 to 69)	–	–	0.0004
Total cholesterol (mmol/l)	4.3 (3.6 to 4.9)	4 (3.5 to 5.1)	–	–	0.6183
HDL (mmol/l)	1 (0.9 to 1.3)	1 (1 to 1.2)	0 (0, 0.1)	–	0.1749
LDL (mmol/l)	2 (1.6 to 2.4)	2.1 (1.6 to 2.7)	–	–	0.423
Trig (mmol/l)	2.1 (1.7 to 3.7)	2 (1.5 to 2.7)	–	–	0.0713
Total Adiponectin (µg/ml)	2.51 (1.92 to 5.65)	3.50 (2.00 to 7.79)	0.87 (0.53, 1.21)	–	<0.00005
**Adipose Tissue volumes**					
Abdominal VAT (L)	7.2 (2.6)	6.3 (2.1)	–	−11.2 (−15.4 to −2.1)	0.0007
Abdominal SAT (L)	13.9 (4.2)	12.9 (4.1)	−0.9 (−1.3, −0.6)	−6.8 (−12 to 0.2)	<0.00005
VAT:SAT ratio	0.5 (0.4 to 0.8)	0.5 (0.4 to 0.7)	–		0.0386
MR Spectroscopy (^1^H MRS)					
Liver fat (% CH_2_/water)	28 (17 to 43)	21 (7 to 29)	−18 (−19, −9)	−42 (−59 to −17)	<0.00005
Soleus IMCL (CH_2_/creatine)	19 (10 to 25)	17 (10.1 to 22)	–	−5 (−31 to 46)	0.4937
Tibialis Anterior IMCL (CH_2_/creatine)	15 (11 to 25)	13 (9 to 21)	–	0 (−38 to 38)	0.8671

* Used natural log of variable pre & post; change is the ratio (post to pre) of geometric means with 95% confidence interval. **Used Wilcoxon matched-pairs. Mean ± standard deviation, median (lower quartile to upper quartile), mean difference (lower 95% confidence limit, upper 95% confidence limit).

### Correlation of Baseline Characteristics with Intrahepatic Lipid

#### Biochemistry

Although many patients had liver biochemistry (ALT, AST and GGT) within the normal reference range, there were highly significant correlations between IHCL and serum levels of ALT (ρ = 0.47), AST (ρ = 0.49), and GGT (ρ = 0.50) (all p<0.05). There was no significant correlation between IHCL and total cholesterol (ρ = 0.16; p = 0.44), LDL-cholesterol (ρ = 0.11; p = 0.64) and triglycerides (ρ = 0.12; p = 0.58)), nor between IHL and total serum adiponectin concentration (ρ = −0.04; p = 0.88).

#### Body composition

There was no significant correlation between IHL and any of the following: body mass index (ρ = 0.16; p = 0.44), total body weight (ρ = −0.24; p = 0.25), total abdominal body fat (ρ = −0.04; p = 0.84), and abdominal visceral (ρ = 0.01; p = 0.96) and abdominal subcutaneous adipose tissue volume (ρ = −0.04; p = 0.86).

### Changes in Metabolic Parameters, Weight and Body Composition with GLP-1 RA (Table 1)

#### Body weight

Six months of treatment with GLP-1 RA (either exenatide or liraglutide) was associated with a significant median weight loss of 5 kg, a relative reduction of 4.3%.

#### Biochemistry

Treatment was associated with significant median reductions in HbA_1_c of 1.6%, in ALT from 40 (31,44) to 31 (27,43) U/L and GGT from 69 (34,100) to 43 (22, 69) U/L. Serum total adiponectin concentration increased significantly in all subjects (p<0.00005). However, a lack of significant correlation between adiponectin increase and any measure of body composition is consistent with previous reports [33].

#### Body composition

There were significant reductions in abdominal VAT and SAT and in IHL. The median relative reduction in IHL was 42%, 4% in total body weight and 7–11% in abdominal SAT or VAT; IMCL did not change.

### Variables Associated with Reduction in Hepatic Steatosis

#### Biochemical response

**Figure 1:**
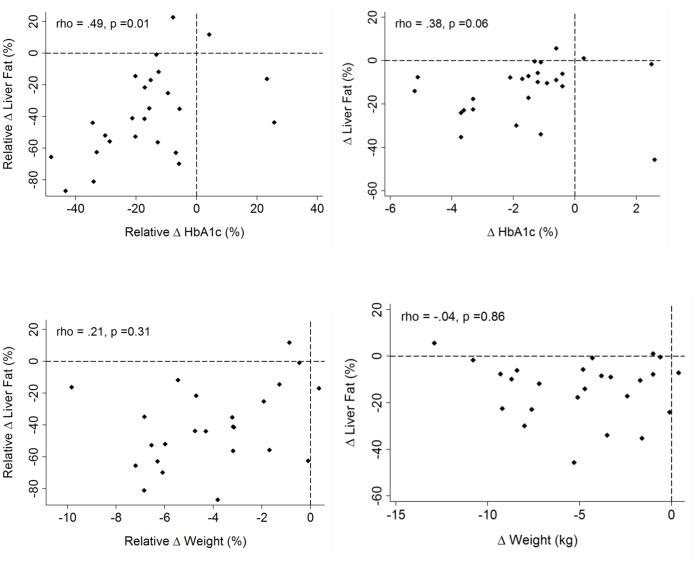
*Upper:* Relative changes in liver fat related to relative change in HbA_1_c (ρ = 0.49; p = 0.01) (left) and absolute change in liver fat related to absolute change in HbA_1_c (ρ = 0.38; p = 0.06) (right). *Lower:* Relative changes in liver fat related to relative change in weight (ρ = 0.21; p = 0.31) (left) and absolute change in liver fat related to absolute change in weight (ρ = −0.04; p = 0.86) (right).

The reduction in IHL with treatment was accompanied by a significant reduction in ALT (p<0.05) and GGT (p<0.001) but not in AST (p = 0.1). There was a significant correlation between relative IHL reduction and relative reduction in HbA1c percentage (ρ = 0.49,p = 0.01); the correlation between absolute changes fell just short of statistical significance (ρ = 0.38,p = 0.06), not surprisingly given the wide range of starting values of IHL, and the strong relationship between the starting IHL and the absolute reduction in IHL (see below). There was no significant correlation between the absolute changes in total adiponectin concentration and IHL (ρ = −0·19, p = 0·43) (Figure 1).

#### Weight loss

**Figure 2:**
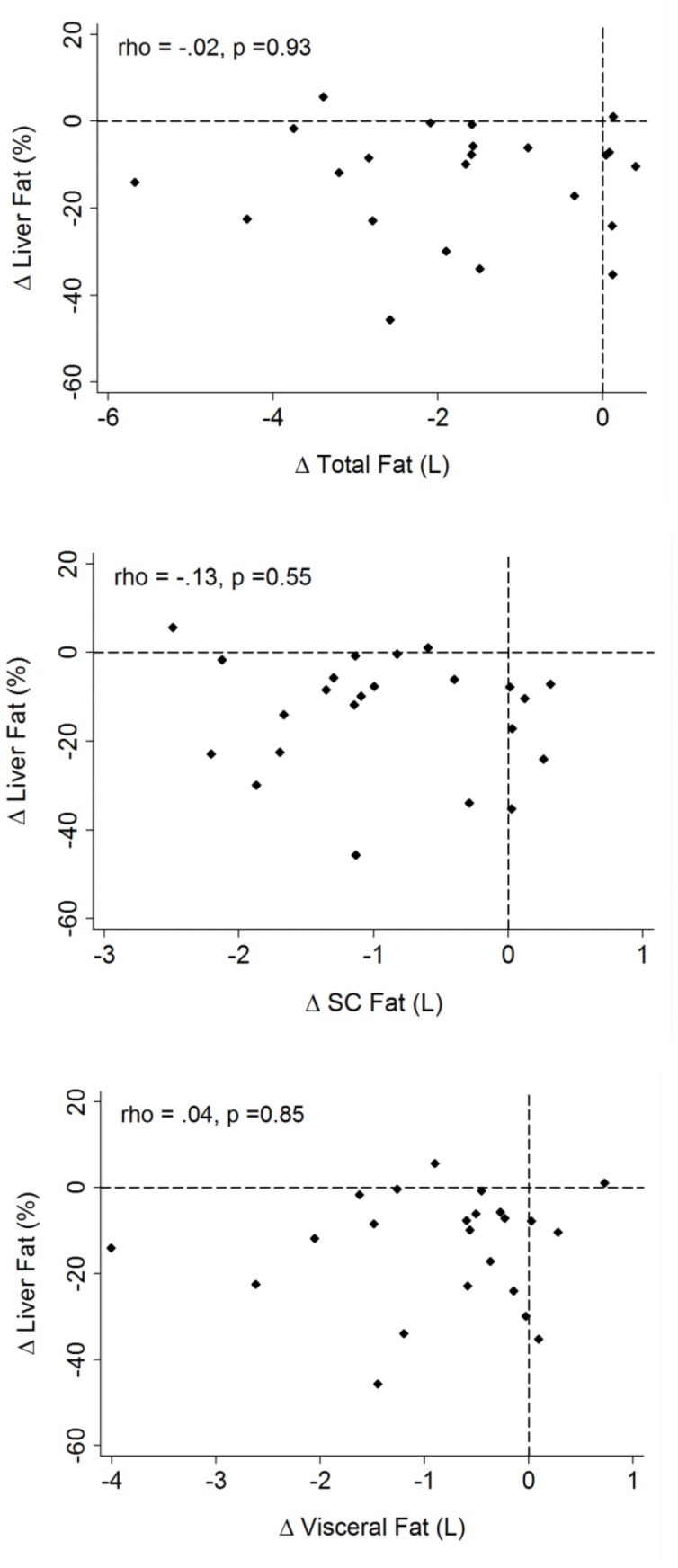
Correlation between changes in liver fat (%) and: *Upper:* change in total abdominal fat (litres). *Middle:* change in abdominal subcutaneous fat (litres). *Lower:* change in intra-abdominal visceral fat (litres).

**Figure 3:**
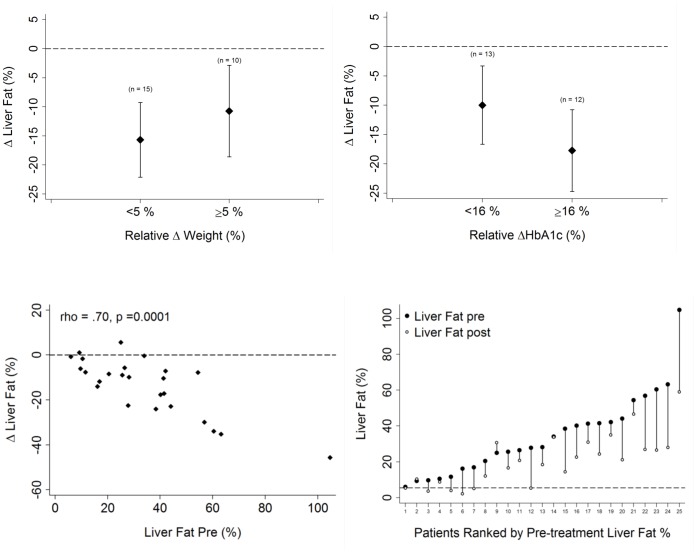
*Upper* Change in liver fat according to weight loss of <5% and >5% and according to relative reduction in HbA1c above or below median (median absolute reduction in HbA1c = 1.3%). *Lower:* Change in liver fat according to pre-treatment liver fat percentage (left) and individual plots to demonstrate changes in liver fat ranked by pre-treatment liver fat percentage (right).

There was no significant relationship overall between the relative or absolute amount of weight loss and the reduction in IHCL (Figure 2). Reinforcing this, when we sub-divided patients into two groups according to amount of weight loss (patients with ≤5% weight loss (n = 15) and patients with ≥5% weight loss (n = 10), the change in IHCL was not significantly different between the two groups (p = 0.35) (Figure 3): there was an absolute reduction in IHL of 15.7% (95% CI: 8.2, 23.2) for patients with ≤5% weight loss (mean weight change, 2.6 (1.5,3.5) kg) versus an absolute reduction in IHL of 10·7% (95% CI 2.7, 18.8) for patients with weight loss of >5% (mean weight change, 8.6 (7,10.3) kg).

#### Abdominal adipose tissue and muscle lipid

(Figure 2) There was no correlation between the reduction in IHCL and the reduction in total abdominal fat (ρ = −0·02; p = 0·93), VAT (ρ = 0·04; p = 0·85), SAT (ρ = −0.13; p = 0·55) or with IMCL in either soleus or tibialis anterior (ρ = −0·17, p = 0·44; ρ = 0·17, p = 0·45 respectively).

#### Baseline intrahepatic lipid

(Figure 3) The most striking correlation was seen between the pre-treatment IHL and the size of the reduction in IHL, such that those individuals with the highest pre-treatment IHL had the greatest absolute reduction in IHL with treatment (r = -0·70; p = 0·0001).

## Discussion

This study demonstrates that 6 months of GLP-1 RA treatment (with exenatide or liraglutide) in patients with T2DM dramatically improves hepatic steatosis. The individual changes in IHL did not correlate with changes in total body weight, and total abdominal fat, VAT or SAT, but it did correlate with reduction in HbA_1c_. A recent randomised parallel-group open-label trial (LEAD-6) reported similar weight loss, but significantly greater reductions in HbA_1c_, using once-daily liraglutide compared with twice daily exenatide [34]. However, we did not observe any differences in these variables between the two agents, albeit using small group sizes.

An obvious question arises from our results: to what extent is reduction in liver fat, mediated by GLP-1 RAs, explained by weight loss? Lifestyle intervention studies have found that modest (5–8%) weight loss has a significant effect on hepatic steatosis in patients with T2DM [21]. Furthermore, Lim *et al*, demonstrate that with a 15% reduction in body weight, with eight weeks of a dietary intervention consisting of a 600 kcal diet, liver fat reduced by a total of 70±5% [35]. However, there has been relatively little work on the effects of GLP-1 RAs on liver fat to address this question. In a single case report, in which 44 weeks of exenatide was associated with a similar reduction in body weight as we observed, a 4 kg absolute reduction or a 5% relative reduction, ^1^H MRS-measured IHCL declined by 73% (from 16% to 4%) [27]. Furthermore, in an uncontrolled study of 8 patients with T2DM and biopsy-proven NAFLD, which examined the effects of 28 weeks exenatide using biopsies pre- and post-treatment, with similar weight loss to that observed in our study (mean weight loss of 4.9 kg), steatosis reduced significantly in half of the patients [28].

In contrast, several reports highlight the differential effects of GLP-1 RAs, and other pharmacological treatments for T2DM, on body composition and IHL. Belfort *et al* examined the effects of 6 months treatment with pioglitazone in 55 patients with either impaired glucose tolerance or T2DM. Treatment was associated with a 54% reduction in ^1^H MRS-measured IHL despite marginal weight gain [25]. The results of other, uncontrolled, studies of glitazone therapy in T2DM demonstrate consistent reductions of ∼50% in liver fat, despite an absence of weight loss or even weight gain, although none have taken paired biopsies to examined for changes in other histological features [23], [36]. In a recent study of 21 patients with T2DM a combination of exenatide with pioglitazone was associated with a greater reduction in ^1^H MRS-measured IHCL than pioglitazone alone (61% vs. 41%), despite there being no overall change in body weight using this pharmalogical combination and weight gain with pioglitazone alone [37]. Likewise, Taikinnen *et al*. demonstrated reductions in hepatic fat with rosiglitazone independent of changes in VAT or SAT [23]. Indeed, in the present study we found no significant relationship between changes in IHL and changes in body weight or any component of fat mass.

There are other potential mechanisms by which GLP-1 RAs may work, perhaps independently of weight loss. GLP-1 RAs may act directly on the hepatocytes. Transgenic rats deficient in dipeptidyl peptidase-4 (DPP4-), the enzyme that degrades endogenous GLP-1, have a threefold higher basal active GLP-1; they not only have lower hepatic fat, but are also protected against hepatic steatosis when fed a high-fat diet [8]. This reduced hepatic fat is mediated by down-regulation of transcription factors for enzymes involved in hepatic lipogenesis and up-regulation of carnitine palmitoyltransferase-1 (CPT1), a key regulator of fatty acid oxidation. Furthermore, *in vitro* studies suggest that the direct action of GLP-1 on hepatic steatosis is mediated by activation of key metabolic signalling pathways, including the AMP-activated protein kinase (AMPK) pathway and the insulin signalling pathway [8], [10], [11], which would be associated with increased hepatic fatty acid oxidation and hence improved insulin sensitivity. The recent identification of GLP1 receptors (GLP1-R) in human liver makes this mechanism a possibility [10]. There have been no randomised controlled studies using GLP-1 RA with paired liver biopsies, for an appropriate time frame, to determine the effects of the GLP-1 RAs on hepatic signalling or metabolic pathways.

The significant increase in serum total adiponectin concentration with GLP-1 RA therapy, consistent with previous data [17], [19], maybe implicated in the mechanism by which liver fat is reduced. However, we did not observe any significant relationship between plasma adiponectin concentration and IHCL, in contrast to a weak correlation (r = −0.219) previously observed in 114 subjects with NAFLD [19].

A further possibility, consistent with the correlation we observed between effects on IHL and HBA_1c_, is that the effects on IHL are secondary to improved glucose tolerance, with reduced hyperinsulinemia (and perhaps reduced hyperglycaemia), reducing the increased rate of hepatocyte lipogenesis characteristic of these patients [13]. The close correlation between changes in IHL and glycaemic control, in absence of changes in other measures of body composition, highlights the key regulatory role of the liver in metabolic control in overweight/obese patients.

Whatever the cause of the change in IHL, the correlation between the pre-treatment IHL and the size of the reduction in IHL is striking (Fig 3): put differently, the fractional change is less variable between patients than the absolute change. It is impossible to exclude the possibility that part of this relationship is due to the purely statistical phenomenon of regression to the mean, but it arguably makes metabolic-control sense that alterations in either downstream or upstream enzymes of fat metabolism should have effects proportional to triglyceride pool size.

It is not clear what effect the reduction in liver fat had on whole body or hepatic insulin resistance in these subjects. Rates of fasting hepatic glucose production (HGP) and insulin sensitivity of liver (assessed by measuring rate of suppression of HGP by insulin) can be determining using [6,6-^2^H_2_] glucose and a hyperinsulinemic, euglycemic clamp [21]. More recently, Hattersley et al. validated a simple index of hepatic insulin resistance using serum glucose and insulin against the gold standard methods using stable isotopes and clamps [38]. For technical reasons, serum insulin concentrations could not be measured in the current study, thus we could not make us of this potentially important physiological measure.

The methodology in this study does not allow us to scrutinize the effects on hepatic inflammation or fibrosis. A longer duration study is warranted with examination of changes in intracellular signaling pathways to assess potential direct mechanisms of action on hepatic lipogenesis and in histopathological changes pre-and post-therapy to address progression through the NAFLD spectrum, including measures of inflammation and fibrosis. Similarly, it is unclear whether these drugs can impact upon the liver-related and cardiovascular burden of NAFLD in these high-risk T2DM patients.

T2DM patients with NAFLD have been suggested as a population worthy of further clinical study to examine the role of insulin sensitizers and related drugs in ameliorating NAFLD [22]. The present findings are thus of potential clinical relevance, and although the present study involves a relatively small sample size and an observational design without use of control subjects, it suggests that these drugs may have a therapeutic benefit, potentially even in lean patients. The literature lacks adequately powered, randomised, placebo-controlled intervention studies using GLP-1 analogues in patients with NAFLD, with or without T2DM, to demonstrate the inter-relationship between the biochemical, metabolic and histological responses. The results of this study should be of use to power a larger, more definitive study incorporating appropriate control groups. This might include a patient group treated with an agent that improves only glycaemic control but not weight (e.g. subcutaneous insulin) and a second group in whom weight loss alone is achieved (e.g. through dietary intervention) to determine the independent contributions of improvements in weight and in glycaemic control. The findings from the present study suggest that the results of such clinical trials using GLP-1 RAs to treat NAFLD might be of considerable therapeutic interest.
